# Spectroscopy (Raman, XPS, and GDMS) and XRD analysis for studying the interaction between nuclear grade graphite and molten 2LiF-BeF_2_ (FLiBe) at 700 °C

**DOI:** 10.1016/j.dib.2018.08.079

**Published:** 2018-08-30

**Authors:** Huali Wu, Francesco Carotti, Ruchi Gakhar, Raluca O. Scarlat

**Affiliations:** University of Wisconsin-Madison, USA

## Abstract

FLiBe-exposed IG-110 graphite and a control IG-110 sample were analyzed by Raman, XPS, GDMS, and XRD, and the complete raw data sets are provided in the Supplementary Information. These data sets enable full reproducibility and transparency of the data analysis we reported in the accompanying research paper titled “Fluorination of Nuclear Graphite IG-110 in Molten FLiBe salt at 700 °C”, published in the Journal of Fluorine Chemistry, and facilitates quantitative comparison with future similar studies by other research groups. In this data article, we provide plots of the peak fitting for all Raman spectra from each sampling point on the graphite surface. We also provide the measured impurity concentrations of the IG-110 samples, as measured by GDMS; this data was not reported nor discussed in the accompanying research paper. The method and software used for peak fitting for the spectra from Raman, XPS, and XRD are listed separately.

**Specifications Table**

**Table:** 

Subject area	Engineering/Nuclear Engineering/Material Science
More specific subject area	Microstructural analysis and elemental analysis of graphite
Type of data	Table, graph, figure
How data was acquired	Raman spectroscopy
	X-ray Diffraction (XRD)
	X-ray photoelectron spectroscopy (XPS)
	Glow Discharged Mass Spectrometry (GDMS)
Data format	Raw data and peak-fitted plots
Experimental factors	Nuclear grade graphite IG-110, and FLiBe-exposed IG-110
Experimental features	Elemental analysis and crystal structure analysis were performed to study the chemical interaction between IG-110 graphite and molten FLiBe
Data source location	Raman Spectra was recorded using a Thermo Scientific DXR Raman microscope in Soft Material Lab (SML), UW-Madison
	XRD diffraction pattern was recorded using a Bruker D8 Discover X-ray Diffractometer in Material Science Center (MSC), UW-Madison
	XPS spectra were recorded using a Thermal Scientific K-alpha X-ray photoelectron spectrometer (XPS) in Material Science Center (MSC), UW-Madison
	GDMS was performed by the EAG laboratories
Data accessibility	Raw data is shared in the Supplementary Information
Related research article	This data article is submitted as a companion paper to a research article: “Fluorination of Nuclear Graphite IG-110 in Molten FLiBe salt at 700 °C”, the research article is submitted to Journal of Fluorine Chemistry [1].

**Value of the data**

• All raw data are presented in this data article as Supplementary Information, which helps the reader to perform their own data analysis with the raw data sets.
• The method used to analyze the data is described in this data article, which helps the reader to reproduce the peak fitting of the XPS, XRD and Raman spectra.
• The full data and data analysis shown in this data article and in the Supplementary Information can be used for quantitative comparison with data from other studies of similar materials exposed to different experimental conditions.

## 1 Data

The interaction between IG-110 and fluoride salt FLiBe (2LiF-BeF2) was studied by immersing graphite samples into molten FLiBe at 700 °C and 1 atm for 12 h. The IG-110 sample was then taken out for elemental analysis (XPS, GDMS) and crystal structure analysis (Raman, XRD). This article reports the detailed data analysis from Raman and GDMS, and a repeatability test for XPS, **as a supplemented data article to the journal paper published in Journal of Fluorine Chemistry** [1] The raw data for Raman, XPS, XRD, GDMS are reported in the Supplementary Information.

## 2 Experimental design, materials, and methods

Control sample of the same shape and similar size was prepared from the same type of graphite. Both the control and test sample were baked under 700 °C for 3 h to remove moisture and gaseous impurities. The IG-110 test sample was characterized together with IG-110 control sample.

Both samples were characterized by Raman spectroscopy, XPS, GDMS and XRD. All spectra from Raman, XPS and XRD were normalized and peak fitted for peak location, peak height, and FWHM by different software and different methods. Peak fitting of Raman normalized spectra were performed in PeakFit v4 software. The software was used to perform data smoothing and background subtraction, then peak fitting with Gaussian function. XRD diffraction data were first analyzed using Diffrac.Eva v3.1 software for stripping the Kα2 component and removing background. The diffraction patterns were normalized and peak fitted by PeakFit v4 software using a deconvolution method. Data processing of XPS spectra were performed by SDP v7.0, a specialized software from PS International. The software is designed for XPS spectra analysis using Gaussian function peak-fitting.

The original data from these characterization techniques are shared as Supplementary Information.

### 2.1 Raman spectra peak fitting

**Fig. 1:**
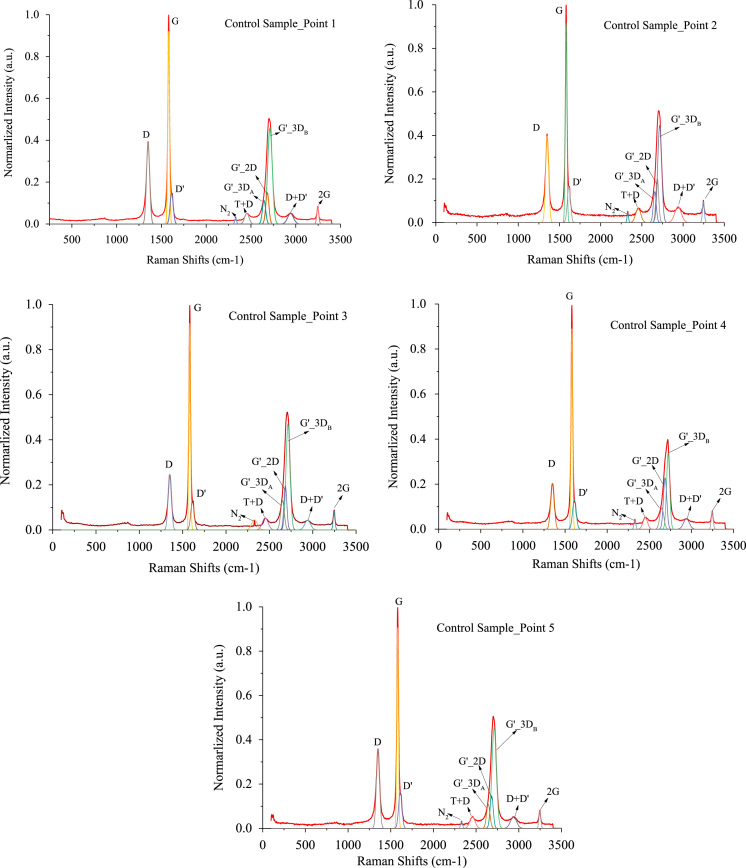
Raman spectra peak fitting plot for control sample (5 points).

**Fig. 2:**
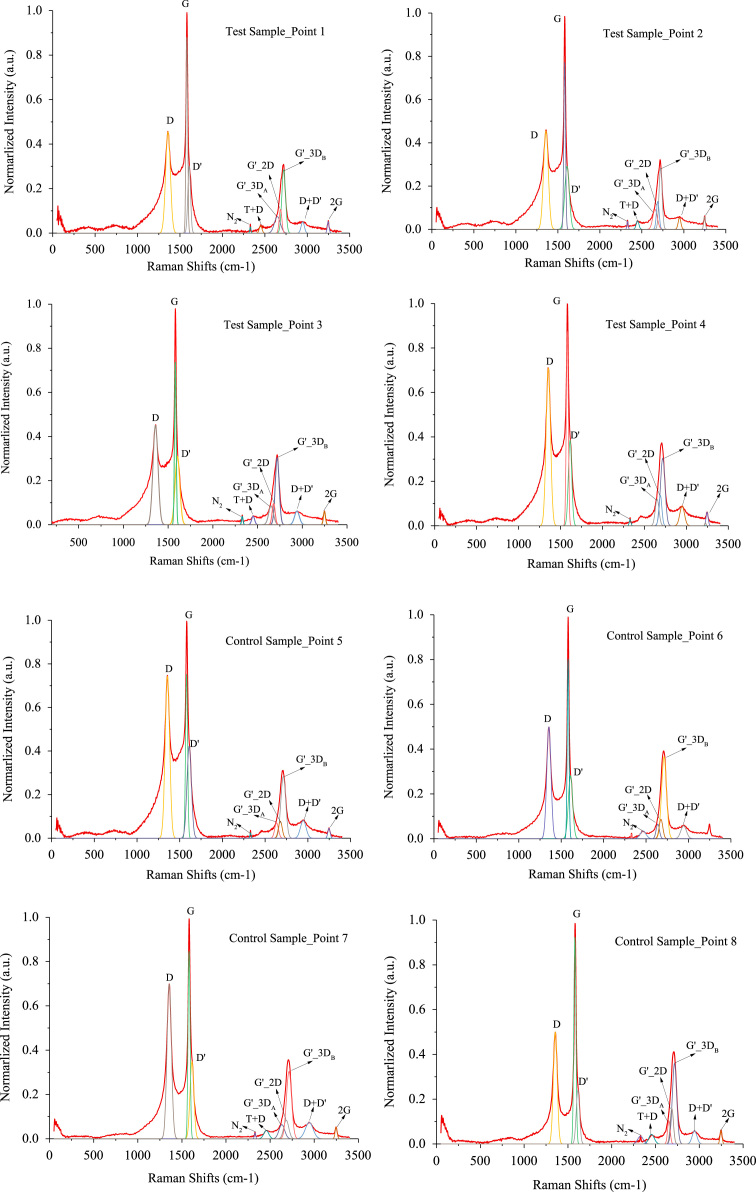
Raman spectra peak fitting plot for test sample (8 points).

Fig. 1, Fig. 2 are the peak fitted Raman data for control sample and test sample. Raman spectroscopy for control sample were performed at five points on the same surface and Raman spectroscopy for test sample were performed at eight points on the same surface. Each spectrum was peak fitted separately. Peak location, amplitude and FWHM were recorded.

### 2.2 GDMS data analysis

**Fig. 3:**
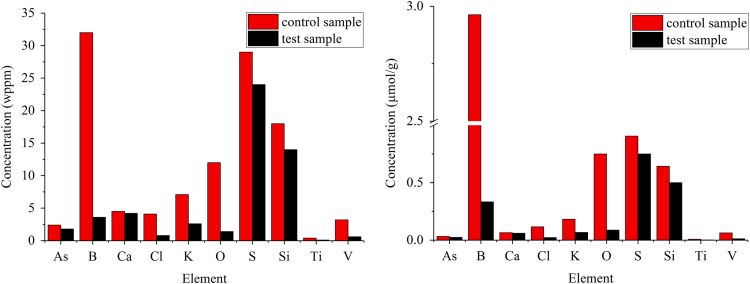
Graphite impurities that decreased in concentration after salt exposure.

**Fig. 4:**
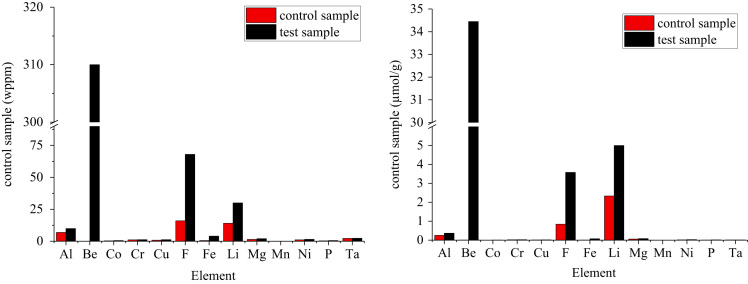
Graphite impurities that increased in concentration after salt exposure.

Fig. 3 shows the impurities in graphite that decreased in concentration after FLiBe exposure, reported in wppm and µmol/g. Fig. 4 shows the elements that increased in concentration after FLiBe exposure, reported in wppm and µmol/g.

## 3 XPS repeatability check (F 1s peak)

**Fig. 5:**
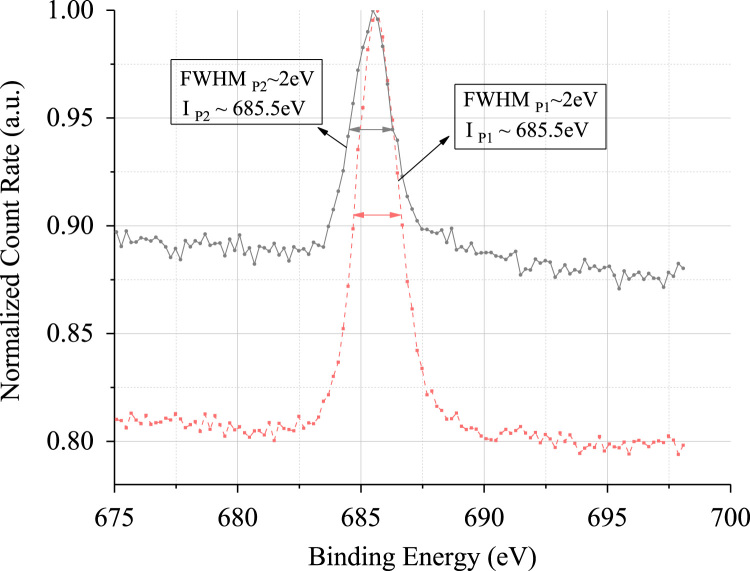
Comparison of F 1s peak at two different points (P1 and P2) on the IG-110 test sample, demonstrating data repeatability.

Fig. 5 is the normalized F 1s spectra from two different points of the surface of test sample. The FWHMs and peak locations of the two spectra are shown in the plot.
