# Supplementary material for: Spectroscopy (Raman, XPS, and GDMS) and XRD analysis for studying the interaction between nuclear grade graphite and molten 2LiF-BeF2 (FLiBe) at 700 °C
Source: Data Brief. 2018 Aug 30;20:1816–21. doi: 10.1016/j.dib.2018.08.079 (PMC6169443; doi:10.1016/j.dib.2018.08.079)
Supplement: Supplementary file 1 — Supplementary material [file mmc1.zip › First page_COI for DIB.PDF.pdf]

Conflicts of Interest Statement

Manuscript title: Spectroscopy (Raman, XPS and GDMS) and XRD analysis for studying the interaction between nuclear graphite and molten 2LiF-BeF2 (FLiBe) at 700°C

The authors whose names are listed immediately below certify that they have NO affiliations with or involvement in any organization or entity with any financial interest (such as honoraria; educational grants; participation in speakers' bureaus; membership, employment, consultancies, stock ownership, or other equity interest; and expert testimony or patent-licensing arrangements), or non-financial interest (such as personal or professional relationships, affiliations, knowledge or beliefs) in the subject matter or materials discussed in this manuscript.

Author names:

Huali Wu

Francesco Carotti

Ruchi Gakhar

Raluca Scarlat

The authors whose names are listed immediately below report the following details of affiliation or involvement in an organization or entity with a financial or non-financial interest in the subject matter or materials discussed in this manuscript. Please specify the nature of the conflict on a separate sheet of paper if the space below is inadequate.

Author names:

Huali Wu

Francesco Carotti

Ruchi Gakhar

Raluca Scarlat
