# Supplementary material for: Spectroscopy (Raman, XPS, and GDMS) and XRD analysis for studying the interaction between nuclear grade graphite and molten 2LiF-BeF2 (FLiBe) at 700 °C
Source: Data Brief. 2018 Aug 30;20:1816–21. doi: 10.1016/j.dib.2018.08.079 (PMC6169443; doi:10.1016/j.dib.2018.08.079)
Supplement: Supplementary file 1 — Supplementary material [file mmc1.zip › second page_COI for DIB.PDF.pdf]

Author's name (typed)	Author's signature	Date
Huali Wu	[illegible]	0[illegible]
Francesco CAROTTI	[illegible]	09[illegible]
Ruchi Gakhar	Ruchi Gakhar	09.[illegible]
[illegible]	[illegible]	[illegible]
